# Vaccination Improves CD4 T Helper Subset Balance in Aged Mice by Overcoming the Effects of the Senescent Environment

**DOI:** 10.1093/geroni/igaa057.441

**Published:** 2020-12-16

**Authors:** Blake Torrance, Erica Lorenzo, Spencer Keilich, Andrew Harrison, Iman Al-Naggar, Laura Haynes

**Affiliations:** UConn Health, Farmington, Connecticut, United States

## Abstract

Influenza (flu) infection is a leading cause of morbidity and mortality in older adults. Although vaccination is the best strategy to combat the effects of flu infection, there is a marked decline in vaccine efficacy in older adults. We hypothesized that an increased level of basal inflammation, a hallmark of the aging, plays a role in this decreased vaccine efficacy and that a vaccination strategy designed to augment the aged response to flu would be beneficial. Adjuvanted vaccination with a conserved influenza protein enhanced viral clearance, antibody production, and protected against weight loss in both young and aged mice. Vaccination also decreased levels of inflammation brought about by leukocyte infiltration and induced a strong type 2 cytokine environment in the lung. Additionally, vaccination reduced the proportion of flu-specific FoxP3+ T regulatory cells (Tregs) and decreased levels of TGF-β in aged lungs following infection. Accumulation of senescent cells, another hallmark of aging, can contribute to the inflammatory environment via their senescent associated secretory phenotype (SASP). Supporting a role for senescent cells in exacerbating age-related deficits in the response to vaccination, treatment of aged mice with the senolytic drugs Dasatinib and Quercetin also decreased levels of Tregs and TGF-β in the lungs following flu infection. These findings give insight to how vaccination responses can be ameliorated in older adults by targeting both inflammatory and regulatory signals and how accumulation of senescent cells could influence the dysregulation of the immune response with age.

